# SPOCK Tool for Constructing Empirical Volcano Diagrams from Catalytic Data

**DOI:** 10.1021/acscatal.5c00412

**Published:** 2025-04-18

**Authors:** Manu Suvarna, Rubén Laplaza, Romain Graux, Núria López, Clémence Corminboeuf, Kjell Jorner, Javier Pérez-Ramírez

**Affiliations:** † Department of Chemistry and Applied Biosciences, Institute for Chemical and Bioengineering, ETH Zurich, Vladimir-Prelog-Weg 1, 8093 Zurich, Switzerland; ‡ Laboratory for Computational Molecular Design, Institute of Chemical Sciences and Engineering, EPFL, 1015 Lausanne, Switzerland; § Institute of Chemical Sciences and Engineering, EPFL, 1015 Lausanne, Switzerland; ∥ The Barcelona Institute of Science and Technology (BIST), Institute of Chemical Research of Catalonia (ICIQ-CERCA), Av. Països Catalans 16, 43007 Tarragona, Spain; ⊥ NCCR Catalysis, 8093 Zurich, Switzerland

**Keywords:** catalyst design, Sabatier principle, linear scaling relationships, machine learning, autonomous discovery

## Abstract

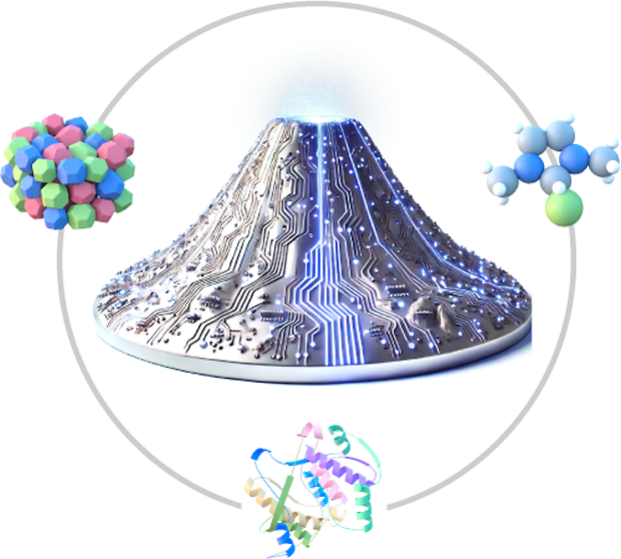

Volcano plots, stemming from the Sabatier principle, visualize descriptor–performance relationships, allowing rational catalyst design. Manually drawn volcanoes originating from experimental studies are potentially prone to human bias as no guidelines or metrics exist to quantify the goodness of fit. To address this limitation, we introduce a framework called SPOCK (systematic piecewise regression for volcanic kinetics) and validate it using experimental data from heterogeneous, homogeneous, and enzymatic catalysis to fit volcano-like relationships. We then generalize this approach to DFT-derived volcanoes and evaluate the tool’s robustness against noisy kinetic data and in identifying false-positive volcanoes, i.e., cases where studies claim a volcano-like relationship exists, but such correlations are not statistically significant. Once the SPOCK’s functional features are established, we demonstrate its potential to identify descriptor–performance relationships, exemplified via the ceria-promoted water–gas shift and single-atom-catalyzed electrocatalytic carbon dioxide reduction reactions. In both cases, the model uncovers descriptors previously unreported, revealing insights that are not easily recognized by human experts. Finally, we showcase SPOCK’s capabilities to formulate multivariable descriptors, an emerging topic in catalysis research. Our work pioneers an automated and standardized tool for volcano plot construction and validation, and we release the model as an open-source web application for greater accessibility and knowledge generation in catalysis.

## 1 Introduction

The Sabatier principle, a foundational concept in rationalizing catalysis, states that a catalyst should bind to a substrate neither too strongly nor too weakly.^1,2^ This behavior manifests in the form of volcano plots and activity maps that describe catalytic performance (e.g., yield, turnover frequency, and overpotential) as a function of a descriptor variable.^3−5^ Ideally, a descriptor should capture information on the catalyst’s active site and local environment and be easy to measure or compute.^4,6^ Typical descriptor variables in heterogeneous catalysis include adsorption energies of reaction intermediates (e.g., H*, OH*, O*, and OOH*) on metal surfaces^3,7,8^ and relative free energies of key catalytic cycle intermediates in homogeneous systems.^9−11^ The success of energy-based descriptors is due to the existence of linear free energy scaling relationships (LFESRs) that connect energies of intermediates and transition states to one (or a few energy contributions, i.e., the descriptors) in the catalytic cycle for a given reaction mechanism within a family of materials due to a similar type of bonding. If these LFESRs exist, then all the energies of intermediates and the transition states linking them are related, and thus, the differential equations describing the rate can be simplified to a central term, the descriptor. The volcano’s peak, indicative of the so-called Sabatier optimal catalyst, corresponds to a situation in which all the reaction steps are balanced, maximizing the overall rate.

The simplicity and straightforward interpretation of volcano plots combined with their quantitative nature have made them pivotal in heterogeneous catalysis since their introduction in the 1950s.^12,13^ In the mid-1990s, developments in density functional theory (DFT) and the pioneering work of Nørskov and co-workers enabled volcano plots’ construction from computational data, significantly expanding their utility.^14−18^ In the past decade, the Sabatier principle has been used to construct volcano plots in homogeneous catalysis, exemplified by research on Suzuki cross-coupling,^19^ hydroformylation of olefins,^11^ and Buchwald–Hartwig amination^20^ reactions, among others. Recently, this concept has been extended to enzymatic transformations.^21,22^ The construction of “first-principles” volcano plots from DFT computations and LFESRs, coupled with microkinetic simulations, depends on the structural model and reaction mechanism assumed. Under these premises, first-principles volcanoes provide valuable mechanistic insights where every step of the catalytic cycle is considered explicitly in the process,^17,18^ and their construction has been largely automated with notable software such as Volcanic,^4^ CatMAP,^23^ and DescMAP.^24^

Manually drawn counterparts stemming from experimental observations, henceforth termed “empirical volcanoes”, present a distinct challenge. Though they are based on chemical knowledge, they are subject to researcher bias and the lack of metrics to quantify the fits. Nonetheless, these plots are vital for identifying reaction descriptors and optimizing reactions with unknown or less studied mechanisms due to the complexity of the reaction or of the material. Data-driven techniques offer alternative strategies for discovering and statistically validating such performance descriptors.^25,26^ Catalysis research has witnessed a surge in interest in adopting machine learning (ML) algorithms such as symbolic regression,^27,28^ SISSO,^29,30^ and subgroup discovery^31,32^ to find single or a combination of descriptors that correlate linearly with performance metrics. While ML-based studies predicting LSFERs are reported,^33−36^ those pertaining to volcano plot construction find sparse mention.^37−39^

**Figure 1:**
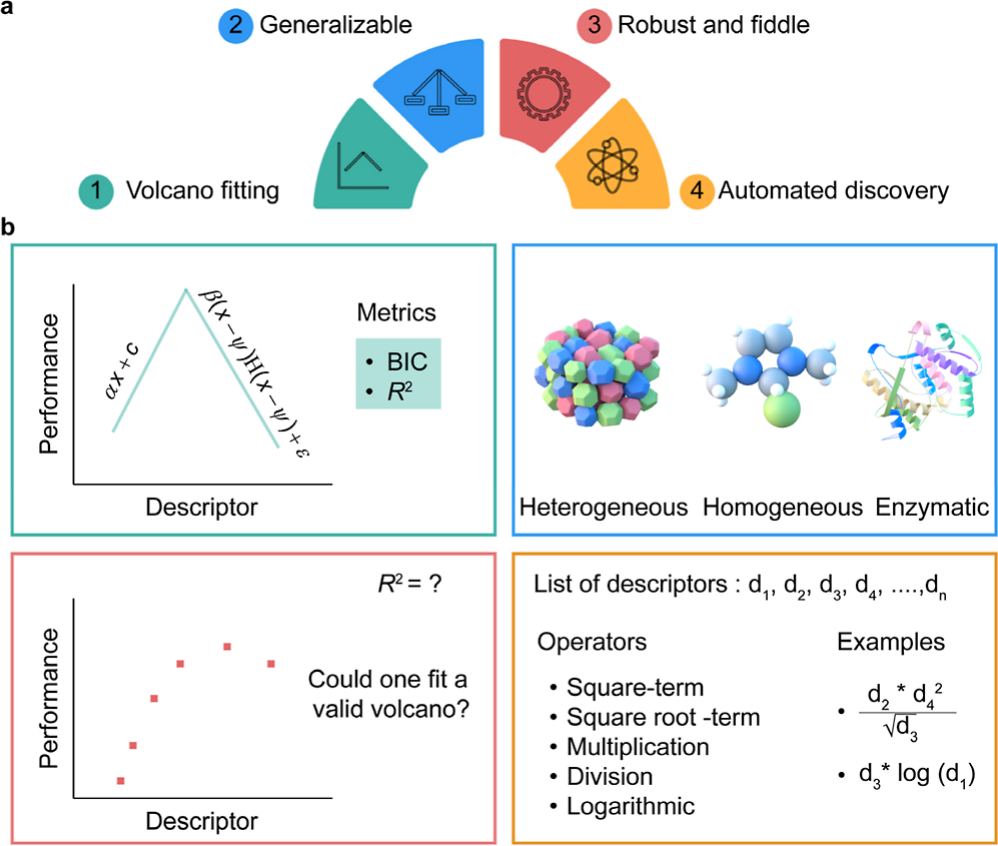
(a) Illustration of SPOCK, highlighting its four essential characteristics. (b) Visual description of each feature to offer a bird’s eye view of SPOCK’s capabilities. The color of the panel border in b corresponds to the SPOCK characteristics depicted in a. Abbreviations used in the figure include BIC: Bayesian information criterion and *R*^2^: coefficient of determination.

Expanding upon these motivations, we introduce SPOCK (systematic piecewise regression for volcanic kinetics), a framework for the automated construction and validation of empirical volcano plots. Essential features of this tool include (i) a standardized and metric-based approach to assist experimental and theoretical researchers alike to construct volcano plots in a consistent and bias-free manner, (ii) generalizable across catalytic chemistries, (iii) robust on encountering noisy kinetic data and ability to invalidate false-positive (FP) volcanoes, and (iv) automated screening and identification of the descriptor–performance relationship, which might be otherwise challenging for the expert practitioner to recognize (Figure 1). Through a series of case studies, we demonstrate each of SPOCK’s features, offering a novel tool for standardized and automated volcano plot construction and a fresh perspective for knowledge generation in catalysis. To maximize the potential of SPOCK, we developed a web application and open-sourced it such that experimental and computational researchers can easily access this tool, requiring no prior programming or coding experience.

## 2 Methods

### 2.1 Piecewise Linear Regression

At the core of SPOCK is piecewise linear regression (PLR), an algorithm that fits multiple linear segments to different ranges of the independent variable and is particularly useful when the relationship between the dependent and independent variables changes at certain points, known as breakpoints. Compared to simple linear regression, PLR must fit the data as well as identify where the breakpoints separate each unique linear segment. Thus, both the breakpoint positions and the equations of each of the linear segments must be fit simultaneously, such that the residual sum of squares is minimized. The mathematical representation of PLR with one breakpoint, which fits two segments, can be written as
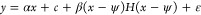
1where given some data *x* and *y* such that *y* = *f* (*x*), α and *c* are the slope and intercept of the first segment, respectively, β is the slope change from the first to second segments at the breakpoint position ψ, *H* is the Heaviside step function, and ε is a noise term.

SPOCK uses a version of the PLR algorithm reported by Muggeo,^40^ which proceeds by fitting the linear segments and refining the position of the breakpoints iteratively. For the mathematical details of the algorithm, we refer the readers to the original publication. Note that the number of fitted parameters in the piecewise equation grows by two for every additional segment. Starting from the classic rule of thumb that requires at least 10 data points to fit a predictive linear model, we consider 10 and 5 observations per segment as the conservative and minimal requirement for PLR, respectively.

Since the Muggeo algorithm requires a predefined number of breakpoints, SPOCK automatically conducts PLR with zero, one, and two breakpoints and selects the best model based on the lowest Bayesian information criterion (BIC),^41^ defined as
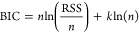
2where RSS is the residual sum of squares, *n* is the number of data points, and *k* is the number of parameters in the final equation.

As increasing the number of segments and breakpoints can yield an artificially improved quality of the fit, i.e., overfit, the *k* ln(*n*) term in the BIC balances the improvement of an additional segment versus the increase in the number of parameters *k*.

### 2.2 Weighting Parameter and Outlier Detection

In SPOCK, each linear fit is performed using weighted least-squares as implemented in the statsmodels python package.^42^ Weighted least-squares, an extension of ordinary least-squares regression, is used when certain data points in the data set are considered more reliable or significant than others. As such, different weights can be assigned to data points, scaling the relative importance or reliability in determining the best-fit based on data trustworthiness. Specifically, in kinetic data, it might be preferred to attribute smaller weights to bad or noisy data points at the bottom of the plot, which may arise due to unforeseen reasons unrelated to the intrinsic catalyst activity (e.g., low stability, solubility issues) or due to unintended error in experimental measurement. We include this option of assigning weights to the data points using the weighting parameter. By varying of the values of this parameter, the model is tuned until a satisfying fit is achieved.

Furthermore, SPOCK uses the EllipticEnvelope method^43^ to detect outliers based on robust covariance estimates. Since no outlier detection strategy is perfect in the low-data regime (e.g., it is very hard to distinguish an outlier from an extreme point), the flagged points are not removed automatically. Instead, SPOCK highlights the points that are likely to be outliers, which can then be double checked by the user and manually removed if required.

### 2.3 Feature Engineering for Descriptor Optimization

SPOCK uses principles of feature engineering by employing simple mathematical operators, i.e., multiplication, division, square term, square root, and logarithm, to augment the provided feature pool and generate combined terms. All of the original and augmented features are then comprehensively screened to find the best possible volcano fit measured in terms of coefficient of determination (*R*^2^), given by the following equation.
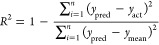
3where *y*_pred_ and *y*_act_ are the predicted and true values of target variable respectively, while *y*_mean_ is the average target variable and *n* is the total number of data points.

### 2.4 Defining the Scope of SPOCK

We examined SPOCK’s ability to construct volcano plots under various scenarios, including standardized data fitting, generalization across homogeneous, heterogeneous, and enzyme catalysis, robustness to erroneous kinetic data, and fidelity against FP volcanoes. After establishing user confidence by evaluating the aforementioned primary features, we used SPOCK to identify novel, physically interpretable, and data-driven descriptors for published studies that lacked such descriptors. A concise overview of the case studies is provided below, with a detailed description of the data compilation process available in Note S1.

### 2.5 Data Sets for Standardized Fitting and Generalization

To evaluate the standardized fitting and generality of SPOCK, we examined four data sets spanning heterogeneous, homogeneous, and enzymatic catalysis subdisciplines. For heterogeneous catalysis, we analyzed thermocatalytic formic acid synthesis,^44^ with the reaction rate mapped against the heat of formation of formic acid (Δ*H*_formate_/kcal mol^–1^) across different temperatures, using data from 12 different metals (Table S1), and the electrocatalytic hydrogen evolution reaction (HER),^45^ mapping current density (Log *i*_0_/A cm^–2^) with the surface energy (*E*_surf_/J m^–2^) for 12 transition metals and metal carbides, resulting in 12 data points (Table S2). Enzymatic catalysis was represented by cellulose degradation, where the reaction rate was mapped as a function of enzyme–substrate binding strength over 13 experimental data points^21^ (Table S3). Lastly, homogeneous catalysis was exemplified by a theoretical study on Suzuki cross-coupling of olefins using 36 metal–ligand complexes,^18^ resulting in an equal number of data points for the analysis (Table S4).

### 2.6 Data Sets for Robustness and Fidelity

To validate the reliability and authenticity of SPOCK, we tested its performance with noisy experimental data and FP volcanoes. Kinetic data, often measured in duplicate or triplicate to ensure reliability, can be noisy due to instrumental errors and manual errors. Such data require uncertainty estimations, including standard deviations or confidence intervals, for reliable interpretation.^46^ FP volcanoes are studies that claim a volcano-like relationship between a descriptor and a performance metric without statistical validation. For the noisy data sets, we examined studies on enzymatic hydrolysis of synthetic polyester^22^ and a perovskite-catalyzed electro-oxygen reduction reaction,^47^ both containing kinetic data with uncertainty estimations for 8 and 15 unique catalysts, respectively (Tables S5 and S6). For FP volcanoes, we examined a theoretical study on the oxygen evolution reaction (OER) using single-atom catalysts (SACs) mapping activity to a composite descriptor variable ϕ, which included electronic and bulk properties of the metal species^48^ (Table S7). We also examined Ni- and Pd-catalyzed cross-coupling reactions, relating activity to the phosphine ligand’s steric and electronic descriptors^49^ (Table S8).

### 2.7 Data Sets for Descriptor Identification

Aligning with the foundational motivation for the development of SPOCK, we explored its potential for automated descriptor identification, eventually uncovering novel catalytic insights. For this purpose, we focus on studies of CeO_2_-promoted catalysts for the thermocatalytic water–gas shift reaction (WGSR)^50^ and SAC-based electrocatalytic CO_2_ reduction reaction (CO_2_RR).^51^ It is worth highlighting that both studies involved screening various catalysts but the intrinsic properties governing catalyst performance remained unidentified.

The WGSR study using CeO_2_-promoted catalysts systematically evaluated transition metals (Pt, Au, Fe, Co, Cu, and Mn).^50^ We extracted catalyst performance data measured as yield (*Y*) and curated a data set linking intrinsic properties of the metal promoters such as covalent radius (*R*_c_), Pauling’s electronegativity (χ), bond length (*d*), cohesive energy (*E*_c_), and metal–support interaction (MSI) parameters like mixed-metal oxide formation energy (*E*_MMO_) and metal oxide formation (*E*_MO_). Similarly, we investigated the data set on the electrocatalytic CO_2_RR by transition metal (TM)-based SACs anchored on nitrogen-doped carbon (TM-N_*x*_, TM = Mn, Fe, Co, Ni, Cu, and Zn; *x* = 4).^51^ Catalyst performance measured as partial current density to CO (*j*_CO_) was extracted from the study, and a data set was compiled linking intrinsic properties of SACs. These included the average electronegativity of the metal atom and its coordination environment, referred to as the electronegativity of the active site (χ_M_), binding energy of single-metal atoms on supports (*E*_B_), stability energy metal atoms on supports (*E*_s_), and diffusion energy (*E*_A_). Both data sets included 6 unique data points, respectively, corresponding to the catalysts investigated, the descriptor definitions are listed (Table S9), and the corresponding data sets are presented (Tables S10 and S11).

It is worth acknowledging that SPOCK does not calculate descriptor values on its own, but rather, this information must be provided by the user through theoretical simulations, mining literature data, or performing experimental measurements. Importantly, the selected descriptors must correlate or inherit fundamental aspects of the electronic structure of the catalyst for meaningful observation of a volcano-like trend. Herein, we leveraged our domain expertise and utilized readily available descriptors from the open quantum materials database^52^ and relevant publications^53−55^ to compile the catalyst-specific properties for both case studies.

### 2.8 Data Sets for Mutlivariable Descriptor Formulations

Catalytic performance is typically influenced by multiple factors, including the electronic and geometric properties of the active site and its interaction with the surrounding environment.^56,57^ Thus, in several instances, identifying effective descriptors for catalytic systems extends beyond single variable property to include combined or mathematically transformed multivariable properties.^57,58^ To evaluate SPOCK’s ability to discern such complex descriptors, we used data sets from previous studies on multimetallic thermocatalytic higher alcohol synthesis (HAS)^55^ (Table S12) and the electrocatalytic HER over transition metals^59^ (Table S13). The HAS data set containing 19 data points used data-driven methods to formulate a composite descriptor by combining intrinsic catalyst features, while the HER data set comprising 23 data points mapped catalytic activity to a single descriptor based on empirical observations. These data sets were used to demonstrate synergistic effects among multiple properties and identify complex descriptors beyond those proposed in the original publications.

## 3 Results and Discussion

### 3.1 Validating the Fitting Accuracy and Generalization of SPOCK

The performance of SPOCK was evaluated using four distinct case studies,^19,21,44,45^ where each of the published studies served as reference benchmarks. These case studies were selected given the clear and evident presence of a volcano-like relation between the descriptor and performance metrics, thus enabling reliable comparison between the established volcanoes and the SPOCK-generated plot. In each case, SPOCK demonstrated a high degree of accuracy as the plots generated closely mimicked those from the original studies, indicating a strong fit and reliable performance. This analysis represents the first crucial step in establishing confidence in the algorithm’s accuracy.

**Figure 2:**
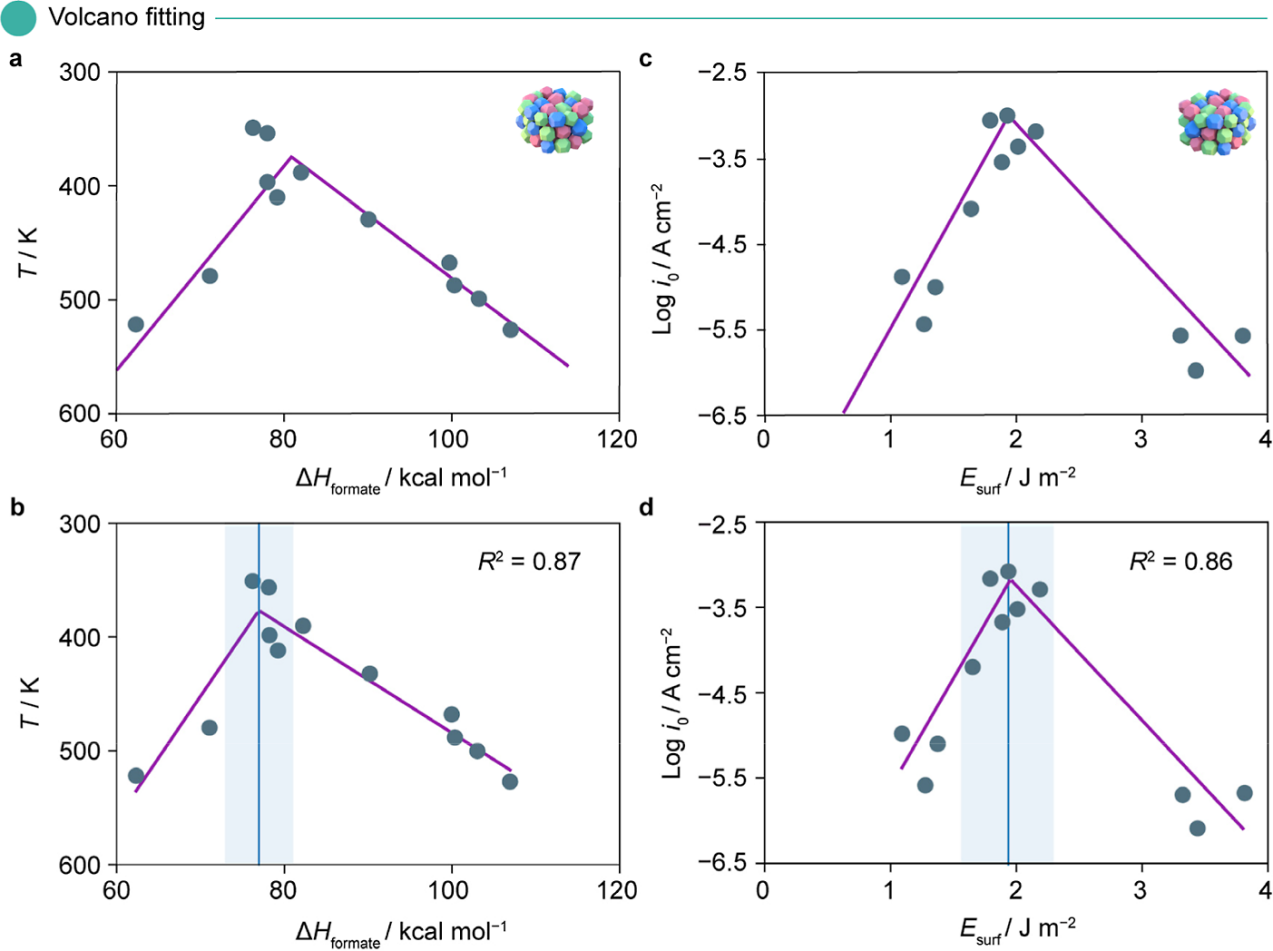
Comparative volcano plots for (a,b) thermocatalytic decomposition of formic acid^44^ and (c,d) electrocatalytic HER,^45^ showing (a,c) for original and (b,d) for SPOCK-generated results. In both cases, SPOCK closely mimics the original trend and provides goodness of fit measured in terms of *R*^2^. The blue line in the SPOCK-generated plots indicates the tip of the volcano, and the shaded region represents the uncertainty around the peak position. The icons used in this plot depict heterogeneous catalysts. Abbreviations with their respective units in parentheses include heat of formation of formic acid (Δ*H*_formate_/kcal mol^–1^), reaction temperature (*T*/K), surface energy of transition metals (*E*_surf_/J m^–2^), and current density (Log *i*_0_/A cm^–2^). Panel (a) is adapted with permission from ref (44). Copyright 1960, De Gruyter. Panel (c) is adapted with permission from ref (45). Copyright 2016, American Chemical Society.

**Figure 3:**
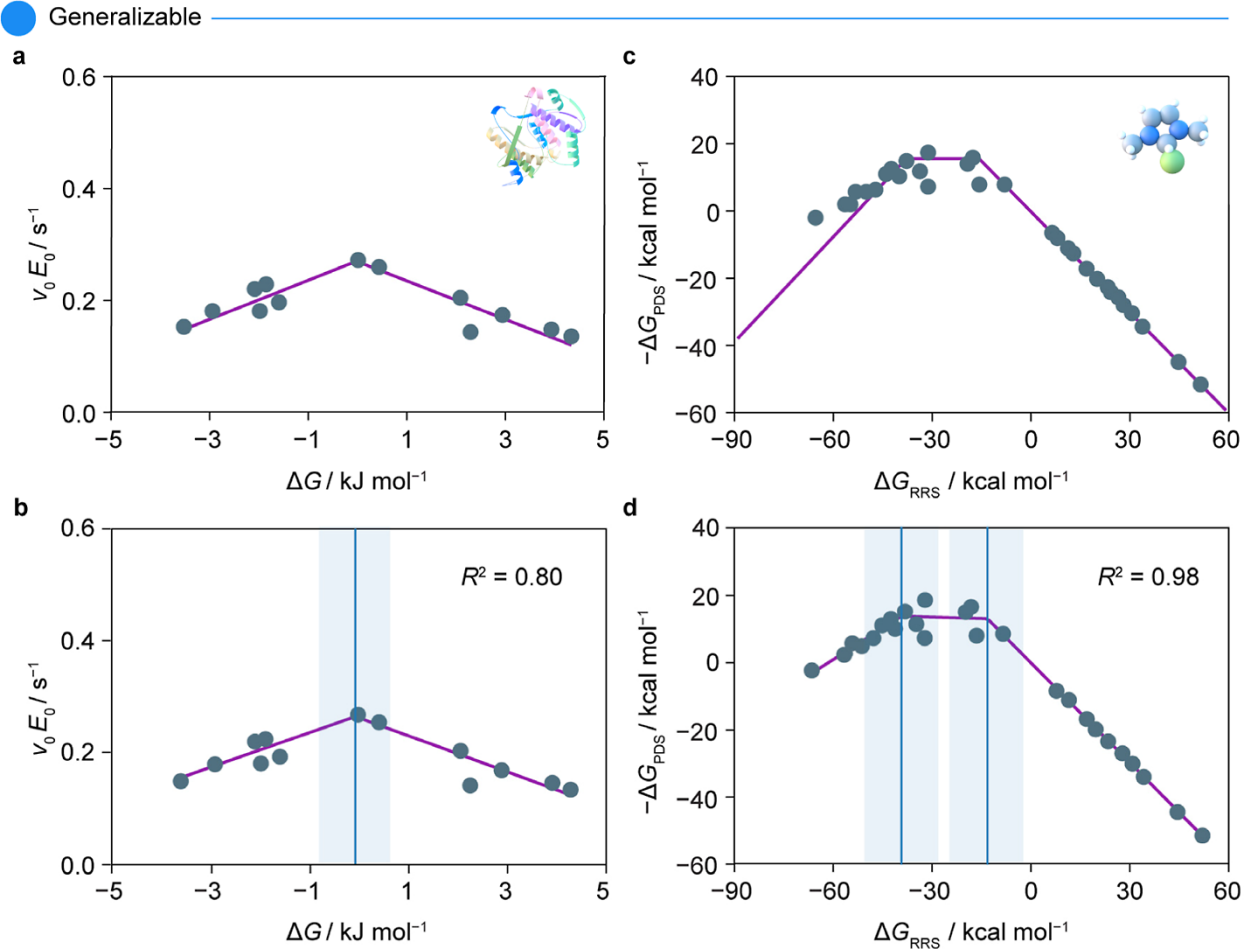
Comparative volcano plots for (a,b) enzymatic degradation of cellulose^21^ and (c,d) theoretical study on Suzuki cross-coupling of olefins over 36 metal–ligand complexes,^19^ showing (a,c) for original and (b,d) for SPOCK-generated results. In both cases, SPOCK closely mimics the original trend and provides goodness of fit measured in terms of *R*^2^. The blue line in the SPOCK-generated plots indicates the tip of the volcano, and the shaded region represents the uncertainty around the peak position. The icons used in the plot represent (a) enzymatic and (c) homogeneous catalysis. Abbreviations used in the plot include enzyme–substrate binding strength (Δ*G*/kJ mol^–1^), reaction rate (*v*_0_*E*_0_/s^–1^), free energy of the relative resting state (Δ*G*_RRS_/kcal mol^–1^), and free energy of the potential determining step (Δ*G*_PDS_/kcal mol^–1^). Panel (a) is adapted with permission from ref (21). Copyright 2018, American Chemical Society. Panel (c) is adapted with permission from ref (19). Available under a CC-BY 3.0 license. Copyright 2015, Busch et al.

For example, in Figure 2a, we reproduce the trend reported in the original publication,^44^ where the activity peak was determined to be around 380 K with an optimal descriptor value of 82 kcal mol^–1^. For comparison, we illustrate the volcano-shaped trend resulting from a SPOCK fit in Figure 2b, where the activity peak was identical to the reference volcano, although the optimal descriptor value is shifted slightly to 77 kcal mol^–1^. The blue line coincides with the volcano’s tip, and the shaded region represents the uncertainty on the peak. Both the right and left slopes of the SPOCK-generated plot are in fair agreement with the original report, with a goodness of fit of *R*^2^ = 0.87. We observe similar performance and trends in applying SPOCK to the original studies of the electrocatalytic HER^45^ (Figure 2c,d) and enzyme-catalyzed cellulose degradation^21^ (Figure 3a,b). Furthermore, SPOCK closely emulates the theoretical volcano trend reported in the Suzuki cross-coupling of olefins over metal–ligand complexes^18^ with a high accuracy *R*^2^ = 0.98 (Figure 3c,d). While the characteristic features of this plot are similar to those previously described, a notable point worth highlighting is the ability of SPOCK to capture the plateau of the volcano, delimited by two breakpoints.

Using diverse data sets from experimental and computational sources, SPOCK consistently delivered high accuracy across heterogeneous, homogeneous, and enzyme catalysis, underscoring its versatility and potential to standardize empirical volcano construction. By standardization, we imply the use of statistical methods, including metrics such as the regression coefficients, root mean squared error, BIC, FP rates, etc., that ensure data fitting reliability and experimental reproducibility. Such best practices have been recently emphasized in ML for chemistry and catalysis^60,61^ and are suitably incorporated in SPOCK, which in turn lay a solid foundation for further testing and validating the model on more complex data sets.

### 3.2 Robustness and Fidelity of SPOCK

**Figure 4:**
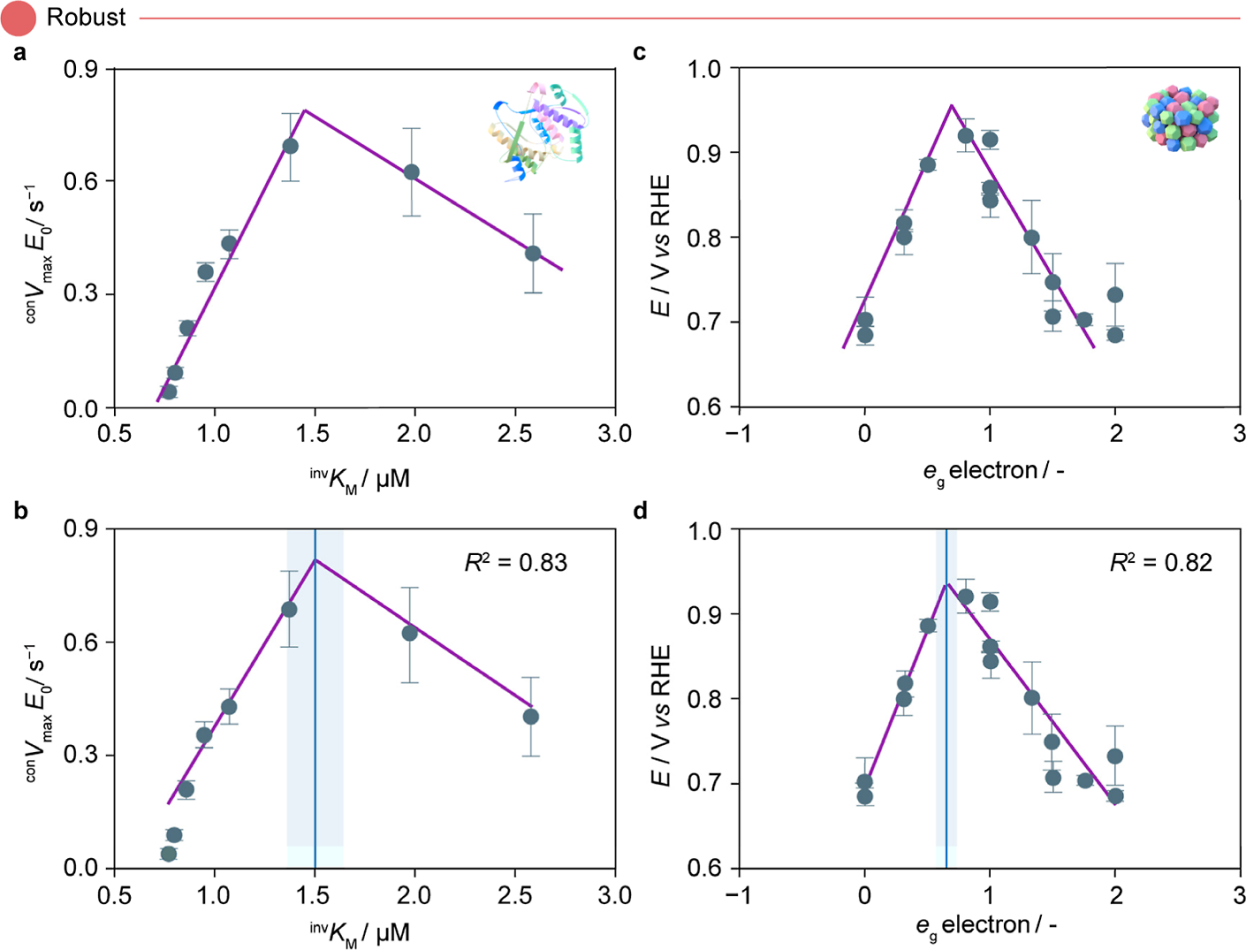
Comparative volcano plots for (a,b) enzymatic hydrolysis of plastics^22^ and (c,d) electrocatalytic oxygen reduction over perovskite oxides,^47^ showing (a,c) for original and (b,d) for SPOCK-generated results. In both cases, SPOCK closely mimics the original trend and provides goodness of fit measured in terms of *R*^2^. The blue line in the SPOCK-generated plots indicates the tip of the volcano, and the shaded region represents the uncertainty around the peak position. The icons used in the plot correspond to those in Figures 2 and 3. Abbreviations used in the plot include the enzyme–substrate binding affinity (^inv^*K*_M_/μM), enzyme saturation condition (^con^*V*_max_*E*_0_/s^–1^), σ*-antibonding orbital filling of transition metal ions (*e*_g_/-), and ORR potential (*E*/*V* vs RHE). Panel (a) is adapted with permission from ref (22). Copyright 2022, American Chemical Society. Panel (c) is adapted with permission from ref (47). Copyright 2011, Springer Nature.

Once the baseline performance of SPOCK was established using relatively straightforward case studies, we evaluated the models’ robustness on encountering data sets with variability and inherent uncertainties. The kinetic data from enzymatic hydrolysis of synthetic polyester^22^ and the perovskite-catalyzed electro-oxygen reduction reaction^47^ contained the mean value of the performance metrics, averaged from duplicate or triplicate experiments, and the respective standard deviations. In Figure 4a, we report the original plot^22^ derived from a heuristic fit following Michael–Menten kinetics and illustrate the SPOCK-derived volcano plot in Figure 4b. In both instances, we observe that the activity peak is close to 0.85 s^–1^, and the optimal descriptor value lies around 0.15 μM. A closer observation reveals that SPOCK fits through the average values in most instances, implying the inherent ability of the model to distinguish between the mean and standard deviation of the data, which is critical for generating accurate plots. Furthermore, a high *R*^2^ = 0.83 is achieved, along with indications of uncertainty in peak estimation, as depicted by the blue shaded region. Similar observations are noticed on evaluating the data set on the electrocatalytic oxygen reduction reaction,^47^ (Figure 4c,d) thereby instilling confidence in the robustness of SPOCK on encountering noisy data.

Empirical volcano plots derived from experimental studies are often susceptible to researcher bias, where perceived correlations between descriptors and performance may reflect human expectations rather than actual data. To this end, we investigated if SPOCK could be used to objectively analyze data sets for the SAC-catalyzed OER^48^ and Ni- and Pd-catalyzed cross-coupling reactions.^49^ In the OER data set, SPOCK initially fit the data with a single breakpoint; however, the statistical significance was low, with *R*^2^ = 0.13 (Figure S1a). Human-based visual inspection also suggested a scattered data distribution rather than a volcano shape. Interestingly, using SPOCK’s outlier detection feature, a single extreme data point at ϕ = 325 units was flagged as an outlier. Upon its removal and subsequent reanalysis, the model fit improved substantially to *R*^2^ = 0.50 (Figure S1b), demonstrating SPOCK’s ability to detect and handle outliers, thereby refining the analysis to discern true patterns from noise. However, in the cross-coupling case study, a negative *R*^2^ was obtained, effectively invalidating any claims of volcano-like correlations (Figure S2). These results highlight the tool’s rigorous evaluation, enabling the differentiation of statistically significant trends from misleading or coincidental ones. By systematically invalidating FP volcano trends and identifying meaningful outliers, the model ensures greater reliability and accuracy in catalysis research. This not only prevents the propagation of erroneous conclusions but also enhances the confidence in empirical data analysis, advancing the scientific rigor of catalyst performance studies.

### 3.3 Descriptor Identification for Catalytic Insights

After demonstrating the basic functional features of SPOCK and establishing user confidence, we evaluated its capability to automate the discovery of single or multiple catalytic properties. Here, the term automation implies SPOCK’s ability to streamline the identification of relevant descriptors through mathematical optimization, eliminating the need for numerous manual fits. The discovery of novel, unreported descriptors underscores SPOCK’s potential to accelerate the identification of descriptor–performance relationships, providing unique catalytic insights. This capability can be harnessed to screen new candidates or explain the empirical behavior of previously studied systems, showcasing the transformative impact of SPOCK in catalysis research.

**Figure 5:**
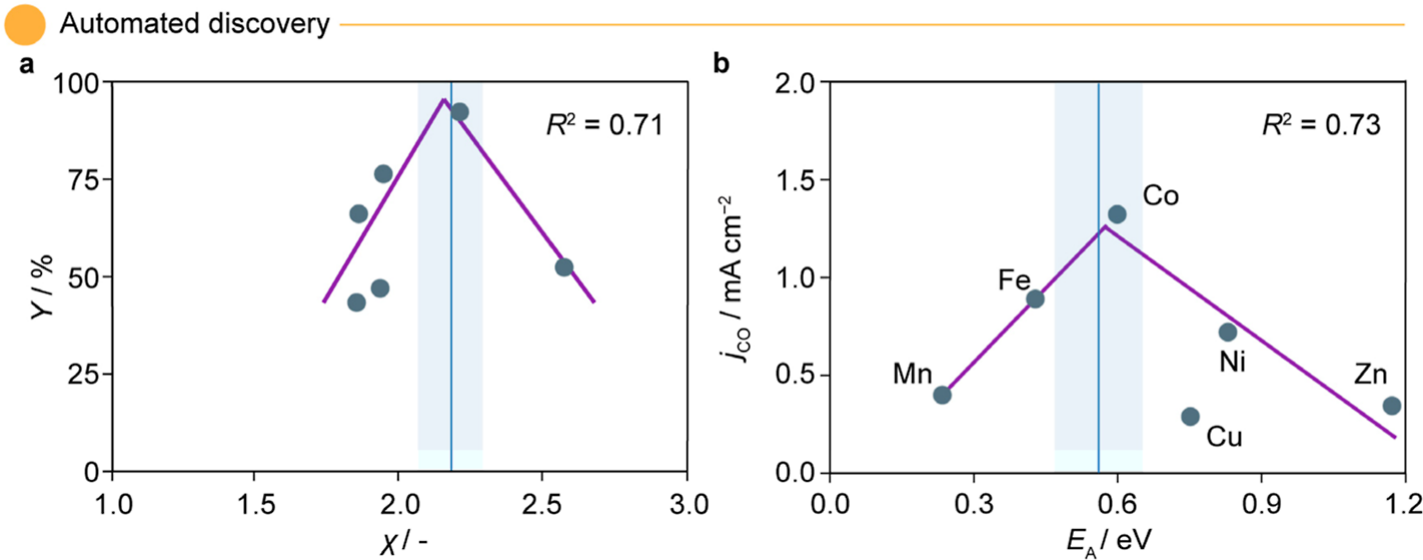
SPOCK-generated volcano plots revealing descriptors for (a) promoted-CeO_2_ based WGSR^50^ and (b) SAC-catalyzed electrocatalytic carbon dioxide reduction^51^ with goodness of fit measured in terms of *R*^2^. The blue line in the generated plots indicates the tip of the volcano, and the shaded region represents the uncertainty around the peak position. Abbreviations with their respective units in parentheses include electronegativity of transition metals (χ/−), WGSR yield (*Y*/%), diffusion activation barrier of the metal atom on the support (*E*_A_/eV), and CO partial current density (*j*_CO_/mA cm^–2^).

We first delve into the thermocatalytic WGSR,^50^ where significant efforts have been dedicated in the past four decades to developing highly active and stable CeO_2_-based catalysts by investigating structure sensitivity, surface acidity and basicity, the promoter effect, and MSI. This catalyst family finds prominence in the WGSR due to the unique role of electronic and geometric effects derived from the synergy of different metal promoters on the CeO_2_ support. While studies investigating MSI for individual metals are reported, no descriptors have been identified to date that explain the empirical performance of a series of promoted-CeO_2_ catalysts. Using SPOCK, we screened 6 descriptors for the WGSR case study, selecting fits with high *R*^2^ and low dimensionality for rationalization. Among the descriptors set, electronegativity of the bulk metals (χ) emerged as a single-variable predictor with *R*^2^ = 0.71 (Figure 5a), which could be correlated to the electronic structure of the materials. Based on these findings, it could be argued that metals with lower χ (e.g., Ni, Fe, Co; χ < 2) facilitate stronger electron donation because of their electronic structures. This characteristic enables the metals to form stronger bonds with adsorbed species, promoting activation of reactants but potentially hindering desorption of intermediates or products. Conversely, metals with χ > 2.5, (e.g., Au) with d*-*bands farther below the Fermi level, could result in weaker adsorbate interactions or weak MSIs, which hamper catalyst activity. Pt with χ = 2.28 demonstrated an optimal balance of adsorbate binding and desorption (2.0 < χ ≤ 2.5), aligning with Sabatier’s principle, balancing strong activation with facile intermediate desorption, as shown in Figure 5a. The emergence of χ as a key descriptor in CeO_2_-promoted catalysts highlights the utility of SPOCK in rationalizing catalytic trends across different metals. Using this descriptor as a guideline, we screen through the 3–5d row elements and identify Ru, Rh, Ir, Pd, and Os as potential metal promoters with 2.0 ≤ χ ≤ 2.5 as alternatives to Pt.

In the electrocatalytic CO_2_RR catalyzed by SACs, the binding energies for CO_2_* and H* are typically attributed as descriptors for CO formation.^51^ Taking a step further in this endeavor, we challenged SPOCK to uncover descriptors beyond traditional adsorption energies. To this aim, we extracted the performance data measured regarding CO partial current density (*j*_CO_) and curated a data set with 4 intrinsic properties of the transition metal atoms. A key requirement for effective supported metal catalysts is maintaining the stability of the active phase, which is dependent on preserving the exposed metal surface. In SACs, stability has usually followed a thermodynamic perspective, defined by the binding of single-metal atoms on supports (*E*_B_), assuming that stronger binding reduces metal atom sintering or agglomeration.^53^ However, a critical factor governing the active sites is the diffusion activation barrier of the metal atom on the support (*E*_A_).^54^ This parameter must be higher than thermal energy to prevent rapid sintering during reactions but not too high such that the metal atoms diffuse into defects in strongly interacting supports, thereby leading to loss of active sites. Based on this fundamental knowledge, we rationalize the observation in Figure 5b, where a volcano-like correlation was observed for *j*_CO_ with *E*_A_ having *R*^2^ = 0.73. For metals like Mn and Zn, the *E*_A_ exhibits high values (>0.8 eV), while for Cu, the values are at the lower spectrum of 0.3 eV. Notably, Ni, Fe, and Co fall in the intermediate levels (0.4 eV ≤ *E*_A_ ≤ 0.8 eV), probing the neither too strong nor too weak assessment with the optimal values of ∼0.6 eV for Co, which resulted in the highest *j*_CO_ = 1.25 mA cm^–2^ in the data set. Therefore, in this case, the descriptor *E*_A_ introduces the aspect of active site number, for which also volcano dependencies have been found.^62^

In summary, both case studies exemplify the powerful capabilities of SPOCK to accelerate the discovery of descriptor–performance relationships, enabling deeper insights into catalytic systems from empirical observations. While the descriptor selection and data curation must be made by a catalysis practitioner, the tool excels at navigating and identifying hidden patterns in data that are challenging for human researchers to discern. By streamlining data analysis and enabling systematic evaluation of descriptors, SPOCK fosters the generation of novel hypotheses and validation of kinetic models, bridging data-driven discovery with chemical intuition.

### 3.4 Toward Complex Multivariable Descriptors

Recent studies, specifically those adopting data-driven methods, have proposed the existence of multiple descriptors that dictate catalytic performance. Catalysis is a complex, multidimensional phenomenon, making it likely that multiple descriptors could affect the reactivity of the catalytic material.^46,56,58,63^ Since SPOCK stems from a data-driven methodology, we showcase its potential to identify multivariable properties, mathematically formulated into a combined descriptor.

**Figure 6:**
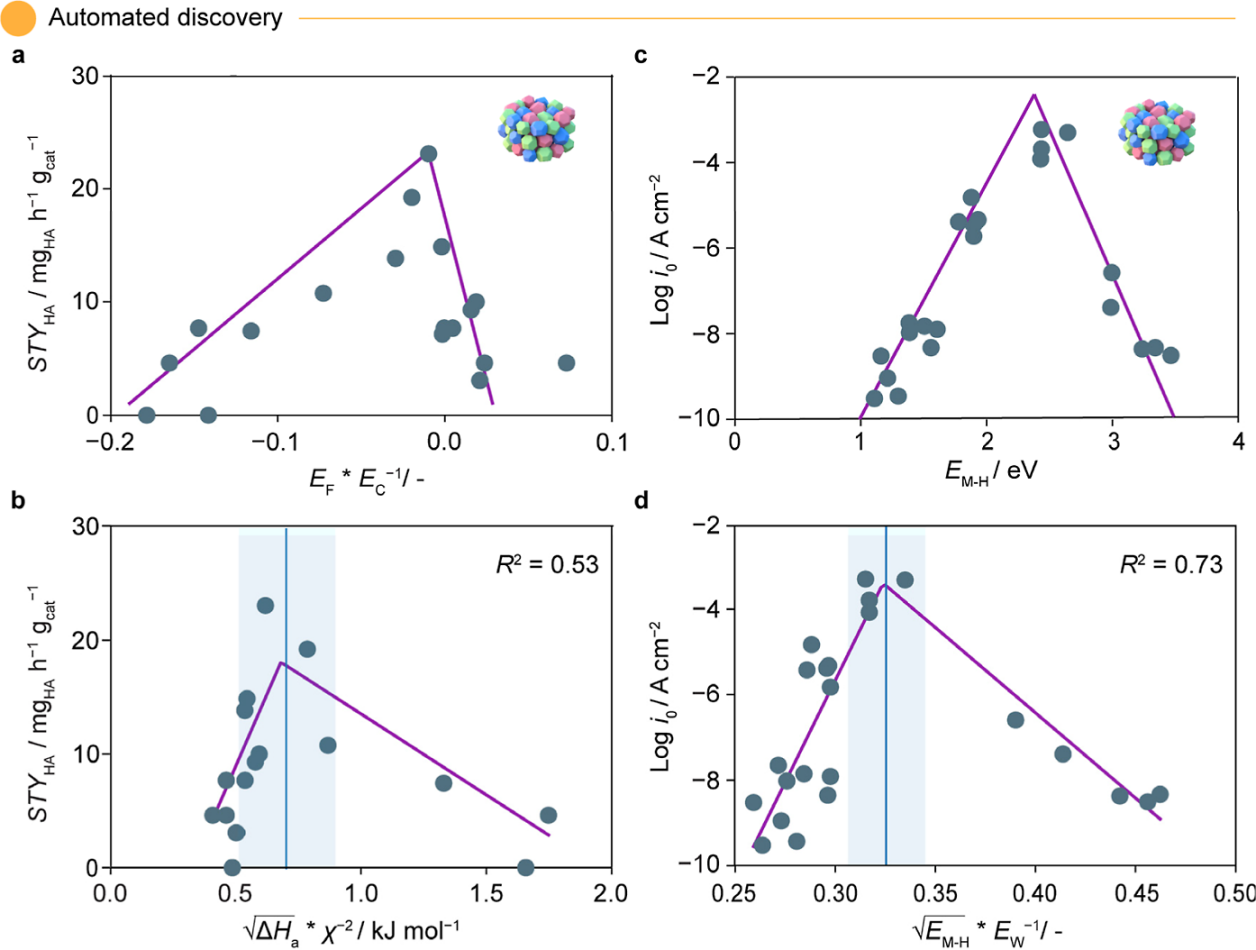
Comparative volcano plots for (a,b) thermocatalytic HAS^55^ and (c,d) electrocatalytic HER,^59^ showing (a,c) for original and (b,d) for SPOCK-generated results. SPOCK enables autonomous discovery of new descriptors for both the reactions with a volcano-like trend. The blue line in the SPOCK-generated plots indicates the tip of the volcano, and the shaded region represents the uncertainty around the peak position. Additionally, the icons used in the plot correspond to those in Figure 2. Abbreviations with their respective units in parentheses include alloy formation energy of transition metals with Rh (*E*_F_/eV), metal cohesive energy (*E*_C_/eV), electronegativity of transition metals (χ/−), enthalpy of atomization (*ΔH*_atom_/kJ mol^–1^), space–time yield of higher alcohols (STY_HA_/mg_HA_ h^–1^ g_cat_^–1^), bond formation energy between bulk metals and hydrogen (*E*_M–H_/eV), work function of metals (*E*_W_/eV), and current density (Log *i*_0_/A cm^–2^). Panel (a) is adapted with permission from ref (55). Copyright 2022, American Chemical Society. Panel (c) is adapted with permission from ref (59). Copyright 1972, Elsevier.

The HAS data set contained several intrinsic catalytic properties describing the performance of Rh-promoted catalysts supported on SiO_2_ to a complex surface descriptor (Figure 6a). On subjecting SPOCK to this data set, two important observations were made, for comparative analysis. First, SPOCK identified the identical descriptor, as reported in the paper, termed promoter affinity index, i.e., the tendency of alkali, transition, and post-transition metal promoters to bind with Rh, the active phase (Figure S3a,b). Additionally, SPOCK discovered a unique descriptor combination that correlates with the target performance with an *R*^2^ = 0.53 (Figure 6b). This included a complex ratio between enthalpy of atomization (Δ*H*_atom_) and electronegativity (χ) of the metal promoters. Δ*H*_atom_ is defined as the enthalpy change associated when metals disintegrate from the bulk phase to dispersed atomic states, while χ indicates the binding tendency of metals. Based on the definitions, the combined descriptor could be rationalized as a measure of metal promoter dispersion over the support, divided by the tendency to bind with Rh or adsorbates, mimicking the surface reactivity of the catalyst.

Similarly, the electrocatalytic HER data set^59^ had identified metal–hydrogen bond formation energy (*E*_M–H_) as a performance descriptor (Figure 6c). On including two additional properties, namely, the work function (*E*_W_) and cohesive energy (*E*_C_) of the metal catalysts, to this data set, SPOCK identified a mathematical formulation between *E*_M–H_ and *E*_W_, resulting in a volcano-like trend with *R*^2^ = 0.73 (Figure 6d). *E*_W_ represents the energy level of the metal, such that surfaces with lower *E*_W_ tend to donate electrons easily, making them more reactive toward electron-accepting adsorbates, while surfaces with higher values attract and stabilize electron-donating species. In conjunction with the *E*_M–H_, the combined descriptors offer a measure to control the interaction of the H* intermediate on the catalyst surface, a critical determinant for the HER.

Aligning with recent studies, both the case studies in this subsection quantitatively underscore the existence of multiple descriptor–performance relationships and highlight the capabilities of SPOCK to uncover such a combination. Nonetheless, balancing the trade-off between accuracy and interpretability is critical, and user discretion is strongly advised. Here, the human expert’s decision making comes into the picture, ultimately deciding if the descriptors proposed by SPOCK align with chemical and physical principles. Nonetheless, identifying these combinations from the vast possibilities is challenging even for expert practitioners, and our model can accelerate this process by analyzing a curated list of potential descriptors, advancing knowledge generation in catalysis.

### 3.5 Web Application and Descriptor Selection

Based on the demonstrated capabilities, we believe SPOCK’s most profound impact will be within the experimental catalysis community. To facilitate greater accessibility, we developed a user-friendly web application that requires no software installation or programming experience, allowing users with no coding experience to use this tool conveniently. The graphical user interface of this application is accessible online, allowing users to upload data in the .xlsx or .csv format. Note S2 provides detailed instructions for preparing data in a machine-readable format, and Note S3 describes the main features of SPOCK, facilitating catalytic practitioners to use the app based on their specific data set and requirements in a reproducible way. The weighting parameter required to replicate the case studies described in the study is listed in Table S14.

**Figure 7:**
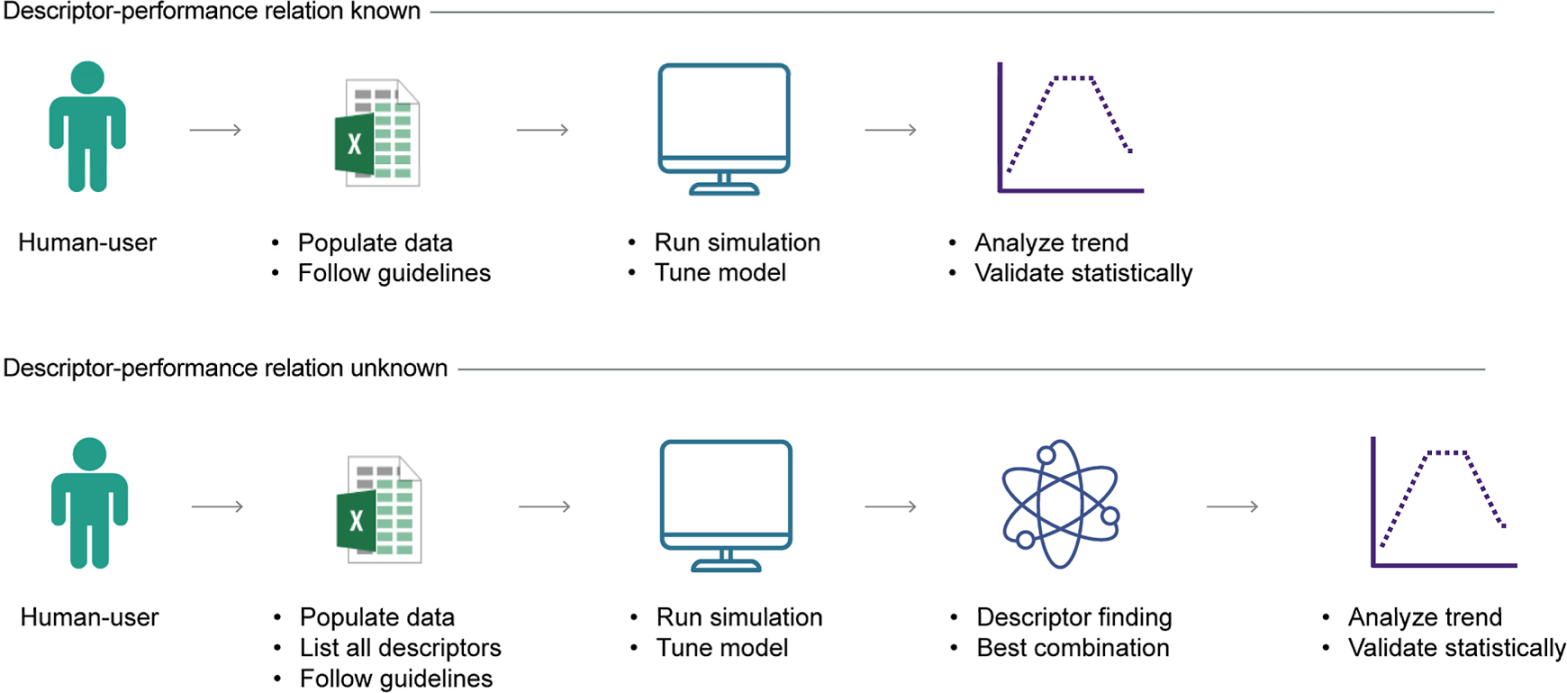
Schematic representation on the user application of SPOCK. The digital tool can be utilized by catalysis researchers in two primary scenarios, and for both instances, the main action steps are listed. By following the guidelines and acknowledging these limitations, users can utilize SPOCK’s features, leading to more reliable and insightful research outcomes.

The web app can be used in two main scenarios. First, if the descriptor–performance relationship is known, then users can upload the relevant data in a machine-readable format and run the simulation. The model analyzes the data, generates a volcano trend, and provides the optimal descriptor value, peak uncertainty, and goodness of fit. Second, if the relationship is unknown, users can list all available descriptors in the input file and run the simulation, whereby possible descriptor combinations will be evaluated and a volcano plot generated if statistically significant correlations are found (Figure 7).

The selection of potential descriptors as inputs to the model is crucial. In heterogeneous catalysis, adsorption energies of reacting species on the catalyst surface are key descriptors across various catalysts and reactions.^3,7,64^ The d*-*band theory introduces descriptors like d*-*band centers and widths, which help understand the impact of the electronic structure of transition metal catalysts on adsorption energies and linear scaling relations.^6,8,57,64^ Additionally, intrinsic catalyst features arising from atomic, bulk, surface, and site-specific properties,^56,58,63^ such as atomic radius, electronegativity, ionization energy, cohesive energy, bond formation energies, coordination number, work function, porosity, etc., affect adsorption energy. These relationships are not always straightforward and may require nonlinear functions to map the catalytic activity. Nonetheless, it is critical that selected descriptors mimic or resonate with the catalyst electronic structure or structural properties (number of sites) for meaningful volcano trends to appear. In homogeneous catalysis, descriptor selection focuses on factors that balance substrate–catalyst interactions,^9,10^ often aligning with the computed energy of reaction steps. Examples include oxidative addition energy,^37^ binding affinity for neutral or anionic species,^65^ and hydrogen-bond donation propensity,^66^ or geometrical descriptors like interatomic distances and angles.^35^ This list of descriptors is not exhaustive but serves as a starting point for users to input into SPOCK to identify significant descriptor–performance relationships.

### 3.6 Limitations and Best Practices for Using SPOCK

While SPOCK offers valuable features for standardized and automated volcano plot construction, it is important to recognize that this is only an initial effort and has inherent limitations. The tool’s performance heavily depends on the quality of input data, and poor experimental data quality can significantly impact the reliability and capability of the model. Few pertinent issues including inconsistent experimental protocols, biased experiments, minimal variability in input parameters, or small data sets arising for very few kinetic experiments can limit the model analysis or misinterpretation of trends. To avoid these pitfalls, systematic experimental designs with proper documentation, incorporating robust data collection protocols, ensuring replicates, and diversifying experimental conditions, are essential to enhancing reproducibility and improving the overall model reliability. For this purpose, we recommend that the users can adhere to the guidelines in Note S2. Additionally, the model identifies relevant descriptors solely on the basis of their correlation with performance metrics, without incorporating chemical insights that infer causation. Therefore, users are strongly encouraged to input descriptors based on established theories or known heuristics. Furthermore, this approach aids in uncovering descriptors to rationalize catalytic trends but does not explain underlying reaction mechanisms, and its potential for designing new materials with desired performances or properties will require further investigation. Understanding the mechanism requires complementary efforts such as DFT, microkinetic simulations, or characterization techniques. It is worth acknowledging that using linear scaling relationships with computed intermediate and transition state energies, as done with tools like Volcanic^4^ or CatMAP,^23^ can offer significant advantages. These include insights into the rate-limiting reaction step and the ability to identify outlier species that may indicate changes in the mechanism or spin state. On a related yet distinct note, it is beyond the current scope and capacity to predict multidimensional volcanoes or those used to capture the trend of multiple products.^67,68^ Finally, while there is no limit on the number of input descriptors, practical use suggests that five to six descriptors allow for comprehensive exploration within 30 min. More descriptors require increased computational resources and time, which could be a constraint. By recognizing and understanding these limitations and best practices, users can better leverage SPOCK’s capabilities, leading to more reliable and insightful research outcomes.

## 4 Conclusions

Constructing volcano plots from experimental data traditionally relies on expert intuition and is prone to human bias. To address these limitations, we introduce a framework that standardizes and automates volcano plot construction. Validated through case studies with published data sets, our approach effectively creates standardized volcano plots with error metrics, handles noisy data, and identifies FPs. It excels in feature engineering, discovering relevant and new descriptors to elucidate descriptor–performance relationships across various catalytic chemistries. While acknowledging the model’s inherent limitations, we provide guidelines for its effective use. SPOCK’s features and innovative approach make it an invaluable asset for catalysis researchers. To ensure broad access, we have open-sourced this digital tool as a web application and provided user guidelines.

## Data Availability

The curated data set and the Python codes required for running the SPOCK package and instructions to generate the volcano plots are available at https://github.com/lcmd-epfl/spock. The data set in machine-readable format and codes have also been deposited in the Zenodo database at 10.5281/zenodo.12804608. We release an open-source web application of SPOCK at https://huggingface.co/spaces/nccr-catalysis/volcano-plot.

## Supporting Information Available

Data sources and best practices for using SPOCK, literature data sets investigated, FP volcano illustrations, and visualization of complex descriptors identified by SPOCK (PDF)
