# Supplementary material for: SPOCK Tool for Constructing Empirical Volcano Diagrams from Catalytic Data
Source: ACS Catal. 2025 Apr 18;15(9):7296–307. doi: 10.1021/acscatal.5c00412 (PMC12053834; doi:10.1021/acscatal.5c00412)
Supplement: Supplementary file 1 — cs5c00412_si_001.pdf [file cs5c00412_si_001.pdf]

Supporting Information

SPOCK Tool for Constructing Empirical Volcano Diagrams from Catalytic Data

Manu Suvarna,[a,e] *Rubén Laplaza,*[b,e] *Romain Graux,*[c,e] *Núria López,*[d] *Clémence Corminboeuf,*[b,e]* *Kjell Jorner,*[a,e]* *and Javier Pérez-Ramírez* [a,e]*

[a] Institute for Chemical and Bioengineering, Department of Chemistry and Applied Biosciences, ETH Zurich, Vladimir-Prelog-Weg 1, 8093 Zurich, Switzerland.

[b] Laboratory for Computational Molecular Design, Institute of Chemical Sciences and Engineering, EPFL, 1015 Lausanne, Switzerland.

[c] Institute of Chemical Sciences and Engineering, EPFL, 1015 Lausanne, Switzerland.

[d] Institute of Chemical Research of Catalonia (ICIQ-CERCA), The Barcelona Institute of Science and Technology (BIST), Av. Països Catalans 16, 43007 Tarragona, Spain.

[e] NCCR Catalysis, 8093 Zurich, Switzerland.

*** Corresponding Authors.**

Clemence Corminboeuf: clemence.corminboeuf@epfl.ch

Kjell Jorner: kjell.jorner@chem.ethz.ch

Javier Pérez-Ramírez: jpr@chem.ethz.ch

Table of Contents

Supplementary Notes…………………………………………………………………………...2

Supplementary Tables ………………………………………………………………….........5

Supplementary Figure ………………………………………………………………….......20

Supplementary References ………………………………………………………………..23

Supplementary Notes

Supplementary Note S1 | Data collection

To compile the data needed for this study, we performed a systematic literature search focused on homogeneous, heterogeneous, and enzymatic catalysis. We utilized a combination of keyword-based search a manual screening. Specifically, we used the keywords (“volcano plots” AND “heterogeneous catalysis” OR “homogeneous catalysis” OR “enzyme catalysis”) to search the Web of Science database, which yielded 86 reports. From these, we manually reviewed and shortlisted 12 publications that were essential for developing the case studies in this work.[1–12] Our selection process was unbiased, ensuring impartial acceptance of data from these publications. The catalytic data necessary to create the volcano plots were primarily extracted directly from tables in the manuscripts or their supporting information. In instances where data were not available in tabular form, we used the open-source WebPlotDigitizer software[13] to extract data from figures within the manuscripts.

Supplementary Note S2 | Data curation for using the SPOCK web app

To effectively populate an Excel sheet (.xlsx or .csv format) to upload on the SPOCK web app, we recommend the following practices to ensure the data is curated and organized properly. Begin by placing the performance metric (target variable) in the first column. Ensure the column is labeled and contains only the target values without any missing or erroneous entries. Next, place each descriptor of interest (input features) in the adjacent columns, one variable per column. Label each column clearly and ensure all cells are filled correctly without missing values or placeholders like "NAN" or "NA". All variables, including the performance metric and descriptors, must be numerical values and follow consistent formatting styles and decimal points. Double-check for outliers or anomalies that could skew model training and remove or correct these entries if necessary. In cases where the kinetic data was compiled from duplicate or triplicate experiments, the performance metric will be represented by the average value and standard deviation. In such instances, a single experimental observation needs to be populated over three rows, such the first, second, and third row contains information on the upper bound, mean, and lower bound data, respectively, of the performance metric. The descriptor values corresponding to these observations remain the same. Before proceeding with model training, validate the calculations for the mean and standard deviations by cross-checking with the original raw data and using statistical formulas to ensure accuracy. Document all essential transformations or preprocessing steps in a separate document linked to the main sheet. This documentation helps ensure transparency and reproducibility in subsequent steps of the project. Maintain version control to track changes and updates to the dataset, ensuring long-term reproducibility of results.

Supplementary Note S3 | Guidelines to use the SPOCK web app

The SPOCK web app is meticulously designed to offer an intuitive user experience, enabling seamless navigation and efficient development of descriptor-performance relationships with minimal effort. We have prioritized a user-centric interface to optimize both functionality and satisfaction.

The "weighting parameter" in SPOCK allows users to fine-tune the model by scaling the volcano trendline across data points. For example, it is often advantageous to assign higher weights to data points located at the top (upper section of the plot) while assigning lower weights to those at the bottom (lower section of the plot), as the latter may be influenced by noise or extraneous factors unrelated to catalyst activity (*e.g.*, low stability, solubility issues). The default setting for the weighting parameter is 1; however, we recommend users experiment with values ranging from 0 to 3 and select the model with the highest accuracy, as indicated by R^2. The "verbosity" setting provides comprehensive details of the model fitting process, including BIC estimates for breakpoints (0, 1, and 2), as well as the intercepts, constants for each breakpoint, and corresponding accuracy metrics. The default verbosity level is set to 3, providing a detailed report of the model's tuning parameters. Users can adjust this between 2 and 5 based on their needs. We recommend setting the weighting parameter and verbosity level before running SPOCK. The "plot mode" feature visualizes the volcano trend on catalytic data, with a default setting of 2 showing the peak as a dashed line. Setting it to 3 includes uncertainty estimates, while 1 presents a simplified trend.

The "Prefit" option, when enabled, performs an initial fitting of the optimal model for each descriptor. This feature is useful for obtaining a quick preview of the potential volcano plot, though it may slow down the overall analysis. The "CBMS" feature, when activated, groups data points by name similarity, assigning them consistent color and marker styles. This is particularly beneficial for visualizing catalyst performance when kinetic data includes duplicates or triplicates, ensuring that a single catalyst is represented with uniform color and marker style. When disabled, all datapoints will be styled uniformly.

Supplementary Tables

Supplementary Table S1 | Empirically obtained descriptor-performance data for the thermocatalytic decomposition of formic acid.[1]

Enthalpy of formation of formate / kcal mol^{-1}	Temperature / K
62.32	521.91
71.07	479.78
79.25	411.85
78.23	398.4
82.24	390.22
78.15	356.42
76.22	350.91
90.2	432.31
99.95	468.11
100.34	488.41
103.01	500.25
106.91	527.19

Supplementary Table S2 | Empirically obtained descriptor-performance data for the electrocatalytic hydrogen evolution reaction.[2]

Surface energy / J m^{-2}	Current density / A cm^{-2}
3.31	−5.99
3.19	−5.60
3.69	−5.57
2.06	−3.19
1.81	−2.98
1.66	−3.06
1.88	−3.42
1.76	−3.57
1.52	−4.10
1.24	−5.00
0.96	−4.88
1.15	−5.48

Supplementary Table S3 | Empirically obtained descriptor-performance data for the enzymatic degradation of cellulose.[3]

Reaction rate / s^{-1}	Enzyme-substrate binding strength / kJ mol^{-1}
−3.60	0.15
−2.91	0.18
−1.98	0.18
−1.60	0.19
−2.10	0.22
−1.89	0.22
−0.02	0.27
0.41	0.25
2.05	0.20
2.89	0.17
3.91	0.15
4.28	0.13
2.25	0.14

Supplementary Table S4 | First-principle computed descriptor-performance data for Suzuki cross-coupling of olefins.[4]

Relative resting state / kcal mol^{-1}	Potential determining step / kcal mol^{-1}
−66.47	−2.44
−55.60	2.54
−56.67	2.20
−54.19	5.63
−51.32	4.77
−47.76	7.18
−45.11	10.95
−42.44	12.83
−41.17	9.92
−34.74	11.30
−32.21	7.19
−38.16	15.06
−32.11	18.50
−19.75	14.92
−18.15	16.46
−16.48	7.90
−8.44	8.42
7.78	−8.51
11.55	−11.24
16.96	−16.89
19.66	−19.97
23.44	−23.56
27.76	−27.15
30.82	−30.23
34.24	−34.16
44.51	−44.59
52.07	−51.61

Supplementary Table S5 | Empirically obtained descriptor-performance data for enzymatic hydrolysis of plastics.[5]

Enzyme saturation rate / s^{-1}	Enzyme-substrate binding affinity / μM
0.08	0.04
0.08	0.09
0.09	0.19
0.09	0.21
0.09	0.22
0.09	0.33
0.09	0.35
0.09	0.38
0.11	0.39
0.11	0.43
0.11	0.47
0.14	0.60
0.14	0.69
0.14	0.78
0.20	0.50
0.20	0.62
0.20	0.73
0.26	0.31
0.26	0.40
0.26	0.50

Supplementary Table S6 | First-principle computed descriptor-performance data for electrocatalytic oxygen reduction reaction.[6]

σ* antibonding orbital filling / −	Potential / V vs RHE
0.00	0.68
0.00	0.70
0.00	0.67
0.00	0.73
0.00	0.70
0.00	0.70
0.31	0.82
0.31	0.80
0.31	0.78
0.32	0.83
0.32	0.82
0.32	0.80
0.50	0.89
0.50	0.89
0.50	0.88
0.81	0.94
0.81	0.92
0.81	0.90
1.00	0.92
1.00	0.91
1.00	0.90
1.00	0.86
1.00	0.84
1.00	0.82
1.00	0.86
1.00	0.86
1.00	0.85
1.33	0.84
1.33	0.80
1.33	0.76
1.49	0.78
1.49	0.75
1.49	0.72
1.50	0.73
1.50	0.71
1.50	0.69
1.75	0.71
1.75	0.70
1.75	0.70
1.99	0.77
1.99	0.73

Supplementary Table S7 | First-principle computed descriptor-performance data for electrocatalytic oxygen reduction reaction.[7]

Phi[a] / -	Overpotential / eV
–1.63	15.48
–1.42	46.44
–1.42	25.80
–1.13	47.13
–1.18	36.81
–1.20	22.71
–1.06	7.91
–1.04	59.86
–0.94	71.90
–0.51	97.71
–0.71	133.14
–0.99	100.11
–1.16	119.72
–1.59	218.81
–0.98	325.80

[a] Phi is a dimensionless descriptor obtained as the dot product between the electronegativity of single-atom catalyst and d-electrons of the metal in the bulk phase.

Supplementary Table S8 | Empirically obtained descriptor-performance data for cross-coupling reactions.[8]

$\%V_{bur}$ – 3 Homo-Lumo gap / min – eV	Yield / %
–1.13	2.50
–0.81	5.61
–0.83	8.88
–0.49	10.28
–0.51	11.84
1.61	12.77
1.97	12.00
1.96	14.02
3.73	14.02
–0.53	14.02
3.77	9.82
6.60	9.82
5.55	8.73
8.01	11.07
12.26	11.86
14.39	11.86
13.30	14.66
14.02	13.73
13.64	16.22
27.86	11.57
34.23	13.14
38.50	12.05
24.29	15.14
34.56	16.09
23.91	17.48
22.13	18.25
23.18	19.81
4.76	18.07
1.93	16.98
–0.56	18.22
6.52	19.79
0.48	21.02
0.49	19.78
2.60	21.65
–0.27	25.22
0.43	26.00
4.32	27.25
–0.70	34.09
16.71	29.76
36.22	29.17
33.85	16.56
38.46	16.41

48.01	19.23
47.31	18.14
54.76	18.30
57.61	15.82
54.35	23.75
59.33	22.82
64.63	25.01
72.42	25.49
63.98	17.38
71.42	18.33
75.66	19.89
77.78	20.67
68.63	13.81
82.80	15.24
87.05	15.40
88.81	16.65
94.84	16.81
84.55	17.42
85.95	19.91
86.54	32.52
55.26	41.81

Supplementary Table S9 | Description of the bulk and atomic properties used as inputs for modelling the automated descriptors case studies.

**Properties**	**Symbol**	**Definition**
Covalent radius	R_C	The half distance between the mid points of the nuclei of two identical atoms joined by a single covalent bond.
Bond length	d	The average distance between the nuclei of two adjacent atoms in the metallic crystal lattice.
Pauling's electronegativity	X	A dimensionless quantity that measures the relative tendency of an atom to attract electrons towards itself within a molecule.
Cohesive energy	E_C	The energy required to disassemble a solid into its individual, neutral free atoms at rest and at infinite separation. .
Work function	E_W	The minimum work needed to extract electrons from the Fermi level of a metal across a surface carrying no net charge..
Alloy formation energy	E_F	The energy change associated with the formation of an alloy from its constituent elements in their standard states.
Metal-hydrogen bond energy	$E_{M\text{-}H}$	The energy required for the adsorption of an H* species on the surface of the bulk metal.
Mixed-metal oxide energy	E_{MMO}	The energy change associated with the formation of mixed metal oxides from its constituent elements and oxide in their standard states.
Metal oxide energy	E_{MO}	The energy change associated with the formation of a metal oxide from its constituent elements.
Enthalpy of atomization	ΔH_{atom}	The enthalpy change accompanying the total separation of all atoms in a chemical substance either an element or a compound.
Average electronegativity	X_M	Electronegativity of the metal atom and its coordination environment.
Binding energy	E_B	The energy required to embed a metal atom on the nitrogen-doped carbon support.
Stability energy	E_S	The energy required for single metal atoms to remain in single-atom form or conversely the tendency to agglomerate into nanoparticles.
Diffusion energy	E_A	The energy required for metal atoms to diffuse across the carrier.

Supplementary Table S10 | Descriptor-performance data compiled for thermocatalytic water gas-shift reaction.[9,14]

Metal	E_C / eV	X / -	d / Å	E_{MMO} / eV	E_{MO} / eV	Y / %
Au	3.81	2.54	2.88	–2.05	–0.46	47.40
Co	4.39	1.88	2.50	–2.42	–1.41	43.62
Cu	3.49	1.90	2.55	–2.46	–0.95	43.90
Fe	4.28	1.83	2.46	–2.67	–1.91	62.64
Ni	4.44	1.91	2.49	–2.28	–0.89	76.05
Pt	5.84	2.28	2.77	–1.79	–0.93	91.80

Supplementary Table S11 | Descriptor-performance data compiled for electrocatalytic carbon dioxide reaction.[10,15]

Metal	X_m / -	E_B / / eV	E_S / eV	E_A / eV	j_{CO} / mA cm^{-2}
Co	0.62	–1.68	4.05	0.64	1.25
Cu	0.63	–1.03	3.16	0.30	0.20
Fe	0.60	–1.44	3.78	0.48	0.75
Mn	0.51	–1.58	3.58	0.85	0.15
Ni	0.63	–1.87	4.63	0.79	0.55
Zn	0.54	–1.78	0.89	1.17	0.10

Supplementary Table S12 | Descriptor-performance data compiled for thermocatalytic higher alcohol synthesis.[11]

Metal	R_C / pm	E_C / eV	E_F / eV	X / -	ΔH_{atom} / kJ mol^{-1}	STY_{HA} / mg$_{HA}$ h^{-1} g$_{cat}^{-1}$
Ag	1.29	2.95	0.22	1.93	284.00	4.62
Ce	1.15	4.32	–0.77	1.12	419.00	0.00
Co	0.54	4.39	0.08	1.88	425.00	10.00
Cu	0.71	3.49	0.07	1.90	338.00	3.08
Fe	0.69	4.28	–0.04	1.83	418.00	23.08
Ga	0.76	2.81	–0.68	1.81	286.00	3.08
Ge	0.53	3.85	–0.55	2.01	377.00	0.00
In	0.94	2.52	–0.41	1.78	243.00	3.08
Ir	0.77	6.94	–0.01	2.20	665.00	14.87
La	1.36	4.47	–0.74	1.10	423.00	4.62
Li	0.90	1.63	–0.19	0.98	161.00	7.44
Ni	0.70	4.44	0.07	1.91	430.00	9.31
Pd	0.78	3.89	0.09	2.20	378.00	4.62
Pt	0.74	5.84	0.01	2.28	565.00	7.69
Re	0.77	8.03	–0.16	1.90	770.00	19.23
Ru	0.82	6.74	0.03	2.20	643.00	7.69
Sn	0.69	3.14	–0.46	1.96	302.00	7.69
V	0.68	5.31	–0.39	1.63	514.00	10.77
W	0.74	8.90	–0.26	2.36	849.00	13.85

Supplementary Table S13 | Descriptor-performance data compiled for electrocatalytic hydrogen evolution reaction.[12]

Metal	$E_{M\text{-}H}$ / eV	E_C / eV	E_W / eV	Log i_0 / A cm^{-2}
Ag	1.41	2.95	4.30	−7.90
Au	1.88	3.81	4.78	−4.80
Bi	1.53	2.18	4.36	−7.84
Cd	1.23	1.16	4.12	−9.01
Co	1.91	4.39	4.70	−5.36
Cu	1.79	3.49	4.70	−5.40
Fe	1.92	4.28	4.65	−5.70
Ga	1.57	2.81	4.25	−8.34
In	1.31	2.52	4.08	−9.43
Ir	2.44	6.94	4.97	−3.88
Mo	3.00	6.82	4.30	−7.38
Nb	3.25	6.82	4.20	−8.36
Ni	1.95	4.44	4.73	−5.29
Pb	1.18	2.03	4.18	−8.51
Pt	2.46	5.84	5.03	−3.25
Re	2.67	8.03	4.95	−3.28
Rh	2.45	5.75	4.99	−3.65
Sn	1.40	3.14	4.35	−7.76
Ta	3.48	8.10	4.22	−8.50
Ti	3.37	4.85	4.10	−8.32
Tl	1.13	1.88	4.02	−9.52
W	3.00	8.90	4.55	−6.57
Zn	1.62	1.35	4.30	−7.90

Supplementary Table S14 | Weighting parameter (WP) for all the case studies investigated and the figure number of the corresponding SPOCK generated volcano in the manuscript.

Case study	Figure	WP value
Thermocatalytic decomposition of formic acid	2b	2
Electrocatalytic hydrogen evolution reaction	2d	1
Enzymatic degradation of cellulose	3b	1
Homogeneous Suzuki cross-coupling	3d	3
Enzymatic hydrolysis of plastics	4b	2
Electrocatalytic oxygen reduction reaction	4d	2
Thermocatalytic water-gas shift reaction	5b	1
Electrocatalytic CO_2 reduction reaction	5d	1
Thermocatalytic higher alcohol synthesis	SI.3b	1
Thermocatalytic higher alcohol synthesis	6b	2
Electrocatalytic hydrogen evolution reaction	SI.3d	1
Electrocatalytic hydrogen evolution reaction	6d	0

Supplementary Figure

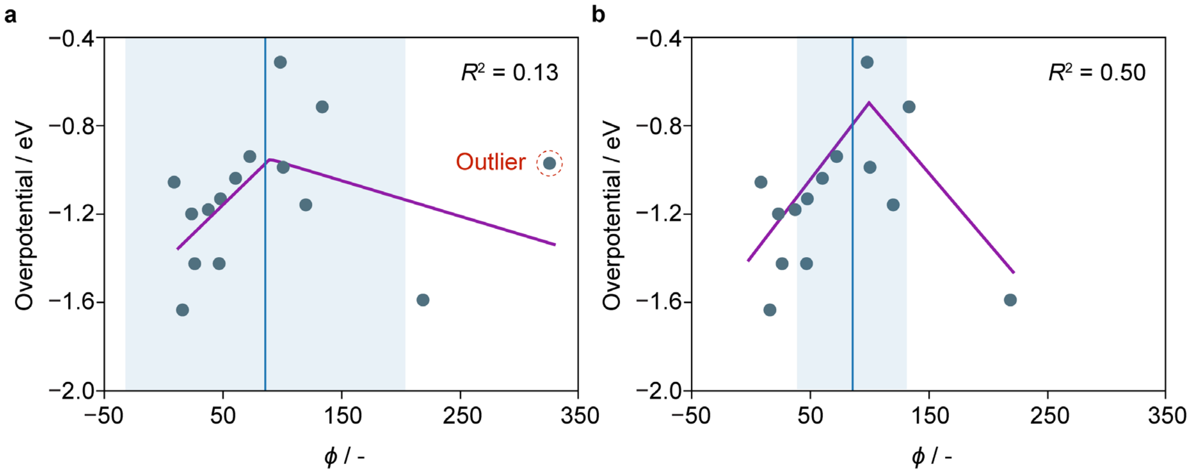

Supplementary Figure S1 | SPOCK-generated plots for **a**) a SAC-catalyzed oxygen evolution reaction[7] An outlier is detected in the dataset. On reanalyzing the dataset after removing the outlier **b**) a significant performance is observed. The blue line in the SPOCK generated plots indicates the breakpoint, and the shaded region represents uncertainty in finding the peak of the volcano.

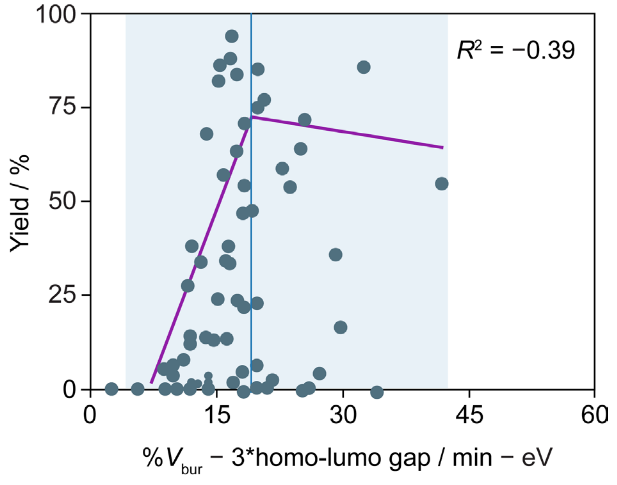

Supplementary Figure S2 | SPOCK-generated plots for organometallic-catalyzed cross-coupling reactions,[8] invalidating the presence of any volcano-like shape between the descriptor and the performance metrics as evidenced by the statistically negative R^2.

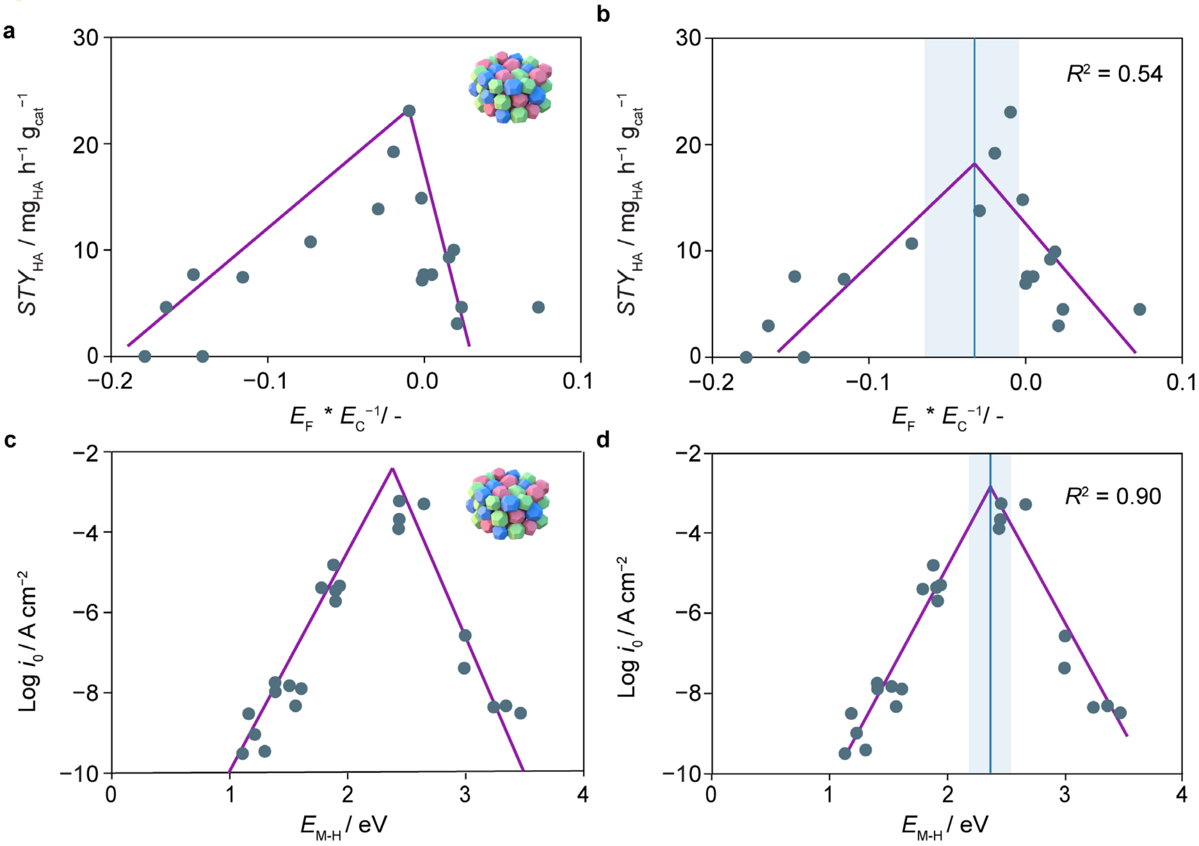

Supplementary Figure S3 | Comparative volcano plots for **a,b**) thermocatalytic higher alcohol synthesis and **c,d**) electrocatalytic hydrogen evolution reaction showing **a, c** for original and **b, d** for SPOCK-generated results**.** SPOCK closely mimics the original trend and provides goodness of fit measured in terms of R^2 between **a-b** and **c-d**.

Supplementary References

(1) Rootsaert, W. J.; Sachtler, W. M. H. Interaction of Formic Acid Vapour with Tungsten. *Z. Für Phys. Chem.* **1960**, *26* (1), 16–26. https://doi.org/10.1524/zpch.1960.26.1_2.016.

(2) Zhuang, H.; Tkalych, A. J.; Carter, E. A. Surface Energy as a Descriptor of Catalytic Activity. *J. Phys. Chem. C* **2016**, *120* (41), 23698–23706. https://doi.org/10.1021/acs.jpcc.6b09687.

(3) Arnling Baath, J.; Jensen, K.; Borch, K.; Westh, P.; Kari, J. Sabatier Principle for Rationalizing Enzymatic Hydrolysis of a Synthetic Polyester. *JACS Au* **2022**, *2* (5), 1223–1231. https://doi.org/10.1021/jacsau.2c00204.

(4) Busch, M.; Wodrich, M. D.; Corminboeuf, C. Linear Scaling Relationships and Volcano Plots in Homogeneous Catalysis-Revisiting the Suzuki Reaction. *Chem. Sci.* **2015**, *6* (12), 6754–6761. https://doi.org/10.1039/C5SC02910D.

(5) Kari, J.; Olsen, J. P.; Jensen, K.; Badino, S. F.; Krogh, K. B. R. M.; Borch, K.; Westh, P. Sabatier Principle for Interfacial (Heterogeneous) Enzyme Catalysis. *ACS Catal.* **2018**, *8* (12), 11966–11972. https://doi.org/10.1021/acscatal.8b03547.

(6) Suntivich, J.; Gasteiger, H. A.; Yabuuchi, N.; Nakanishi, H.; Goodenough, J. B.; Shao-Horn, Y. Design Principles for Oxygen-Reduction Activity on Perovskite Oxide Catalysts for Fuel Cells and Metal-Air Batteries. *Nat. Chem.* **2011**, *3* (7), 546–550. https://doi.org/10.1038/nchem.1069.

(7) Wu, L.; Guo, T.; Li, T. Machine Learning-Accelerated Prediction of Overpotential of Oxygen Evolution Reaction of Single-Atom Catalysts. *iScience* **2021**, *24* (5), 102398. https://doi.org/10.1016/j.isci.2021.102398.

(8) Chen, J.; Zhang, R. Volcano Plots of Reaction Yields in Cross-Coupling Catalysis. *J. Phys. Chem. Lett.* **2022**, *13* (2), 520–526. https://doi.org/10.1021/acs.jpclett.1c04099.

(9) Jacobs, G.; Chenu, E.; Patterson, P. M.; Williams, L.; Sparks, D.; Thomas, G.; Davis, B. H. Water-Gas Shift: Comparative Screening of Metal Promoters for Metal/Ceria Systems and Role of the Metal. *Appl. Catal. Gen.* **2004**, *258* (2), 203–214. https://doi.org/10.1016/j.apcata.2003.09.007.

(10) Li, J.; Pršlja, P.; Shinagawa, T.; Martín Fernández, A. J.; Krumeich, F.; Artyushkova, K.; Atanassov, P.; Zitolo, A.; Zhou, Y.; García-Muelas, R.; López, N.; Pérez-Ramírez, J.; Jaouen, F. Volcano Trend in Electrocatalytic CO_2 Reduction Activity over Atomically Dispersed Metal Sites on Nitrogen-Doped Carbon. *ACS Catal.* **2019**, *9* (11), 10426–10439. https://doi.org/10.1021/acscatal.9b02594.

(11) Suvarna, M.; Preikschas, P.; Pérez-Ramírez, J. Identifying Descriptors for Promoted Rhodium-Based Catalysts for Higher Alcohol Synthesis via Machine Learning. *ACS Catal.* **2022**, *12* (24), 15373–15385. https://doi.org/10.1021/acscatal.2c04349.

(12) Trasatti, S. Work Function, Electronegativity, and Electrochemical Behaviour of Metals: III. Electrolytic Hydrogen Evolution in Acid Solutions. *J. Electroanal. Chem. Interfacial Electrochem.* **1972**, *39* (1), 163–184. https://doi.org/10.1016/S0022-0728(72)80485-6.

(13) automeris.io: AI Assisted Data Extraction from Charts using WebPlotDigitizer. https://automeris.io/ (accessed 2024-07-22).

(14) Kirklin, S.; Saal, J. E.; Meredig, B.; Thompson, A.; Doak, J. W.; Aykol, M.; Rühl, S.; Wolverton, C. The Open Quantum Materials Database (OQMD): Assessing the Accuracy of DFT Formation Energies. *Npj Comput. Mater.* **2015**, *1* (1), 1–15. https://doi.org/10.1038/npjcompumats.2015.10.

(15) Ha, M.; Kim, D. Y.; Umer, M.; Gladkikh, V.; Myung, C. W.; Kim, K. S. Tuning Metal Single Atoms Embedded in NxCy Moieties toward High-Performance Electrocatalysis. *Energy Environ. Sci.* **2021**, *14* (6), 3455–3468. https://doi.org/10.1039/D1EE00154J.
